# Effects of phytosterols on growth performance and meat quality in finishing pigs: role of lipid metabolism and gut microbiota

**DOI:** 10.3389/fvets.2026.1864330

**Published:** 2026-06-23

**Authors:** Songshi Zhong, Chenglong Li, Qiyu Tian, Lingyuan Yang, Liwen Mei, Xingguo Huang, Yinghui Li

**Affiliations:** 1 College of Animal Science and Technology, Hunan Agricultural University, Changsha, China; 2 Key Laboratory of Livestock and Poultry Resources (Pig) Evaluation and Utilization, Ministry of Agriculture and Rural Affairs, Hunan Agricultural University, Changsha, China; 3 Yuelushan Laboratory, College of Animal Science and Technology, Hunan Agricultural University, Changsha, China; 4 Hunan Pig Industry Technological System Research and Development Center, Hunan Agricultural University, Changsha, China

**Keywords:** finishing pig, gut microbiota, lipid metabolism, meat quality, phytosterols

## Abstract

This study aimed to investigate the effects of dietary phytosterol (PS) supplementation on growth performance, meat quality, and lipid metabolism in finishing pigs and to explore the potential mechanisms. Forty-eight barrows with an initial body weight of 73.96 ± 2.80 kg were randomly allocated to two treatments, with six replicate pens of four pigs per pen: the CON group fed a basal diet, and the PS group fed a basal diet with 300 mg/kg of PS for a period of 42 days. Dietary PS improved the average daily gain, apparent digestibility of ether extract, and jejunal lipase concentration (*P* < 0.05). Additionally, PS increased the serum high-density lipoprotein cholesterol level, intramuscular fat concentration, and essential fatty acid concentrations in the muscle, including C18:2n6c, C18:3n3, and n-3 PUFAs (*P* < 0.05). Furthermore, PS upregulated the mRNA expression of genes associated with lipogenesis and transportation, such as acetyl-coenzyme A carboxylase, fatty acid synthase, and peroxisome proliferator-activated receptor γ (*P* < 0.05), and tended to increase the mRNA expression of cluster of differentiation 36 (CD36; *P* = 0.09). Colonic microbiota comparisons showed that dietary PS downregulated the relative abundance of *Clostridium_sensu_stricto_1, Escherichia-Shigella*, and *Prevotella_9*, while upregulating the relative abundance of *unidentified Ruminococcaceae* and *Paludicola*. Correlation analysis indicated that the relative abundance of *Prevotella_9* was negatively correlated with the expression of CD36, whereas *Paludicola* and *unidentified Ruminococcaceae* were positively correlated with the expression of lipogenic genes. In conclusion, PS can increase growth performance, enhance nutrient digestion, and improve pork quality by increasing intramuscular fat deposition in finishing pigs, a process that involves the regulation of the gut microbiota and lipid metabolism.

## 1 Introduction

With socioeconomic development and rising consumer living standards, the demand for tastier, more nutritious, and higher-quality pork is increasing. Over the past few decades, the excessive pursuit of growth rate and leanness by pig producers has resulted in a decrease in meat quality. Hence, improving carcass and meat quality has always

been a focus of attention in the pig industry (1). Meat quality is a composite economic characteristic, and indicators of meat quality include color, pH, and intramuscular fat (IMF) content (2). IMF content is one of the most critical traits influencing meat quality and is closely linked to tenderness, flavor, and water-holding capacity (3). Additionally, muscle fatty acid profiles have a critical impact on meat quality. Previous research has shown that polyunsaturated fatty acids (PUFAs), especially n-3 PUFAs, exert positive effects on health, including the regulation of gut immunity (4) and prevention of cardiovascular disease (5). n-3 PUFA deficiency has been associated with the development of metabolic disorders, autoimmune diseases, and inflammatory diseases (6). Nutritional regulation is a more practical way to improve meat quality than genetic breeding because of the lengthy cycle.

Phytosterols (PS) are natural bioactive compounds obtained from plants that are structurally similar to cholesterol. PS are found in vegetable oils, nuts, and seeds in free, glycoside, and esterified forms (7). Over 250 different PS molecules have been identified, among which β-sitosterol is the most abundant, accounting for 60%−90% of PS (8). Numerous studies have demonstrated that PS have beneficial effects on preventing and treating hypercholesterolemia, cardiovascular diseases, and inflammation (9, 10). PS can compete against cholesterol for intestinal absorption sites, thereby reducing cholesterol concentration in the serum and alleviating steatosis, inflammation, apoptosis, and oxidative stress (11).

Recently, several researchers have explored the use of PS in animal husbandry. Accumulating evidence has shown that PS supplementation can increase growth performance, enhance antioxidant capacity, regulate gut microbiota, and exert beneficial effects on broiler meat quality (12, 13). Previous studies have also demonstrated the benefits of PS supplementation on the growth performance and health of pigs. For instance, PS supplementation could lower serum cholesterol and improve intestinal morphology, ultimately contributing to a decreased diarrhea rate and improved health in weaned piglets (14). However, reports regarding the effects of PS supplementation on pork quality are scarce. Given the close association between lipid metabolism and meat quality traits, further elucidation of the effect of PS on lipid metabolism will help to better understand its role in pork quality. Based on the above, this study was conducted to investigate growth performance and meat quality in finishing pigs supplemented with PS and to elucidate its molecular mechanism from the perspective of lipid metabolism and gut microbiota, thereby providing a theoretical foundation for PS application in finishing diets.

## 2 Materials and methods

### 2.1 Ethical statement

The experimental procedures performed in the animal experiments were authorized by the Animal Welfare Committee of Hunan Agricultural University (Protocol code 2022123).

### 2.2 Animals, diet, and experimental design

Forty-eight barrows (Duroc × Large White × Landrace, initial body weight of 73.96 ± 2.80 kg) were randomly allocated into two treatments, with six replicate pens of four pigs per pen. Pigs were fed the basal diet (CON) or the basal diet supplemented with 300 mg of phytosterols/kg (PS). PS were derived from soybeans, with 95.00% purity, containing β-sitosterol (50.93%), campesterol (29.92%), stigmasterol (18.26%), and brassicasterol (0.98%). The PS dosage used was based on previous studies that showed improvements in growth performance and increased economic benefits in pigs (15). The basal diet was formulated as a powder and designed to meet all nutrient requirements recommended by the National Research Council ((62); Table 1). After a 3-day adaptation period, pigs were housed in pens with concrete slatted floors and had *ad libitum* access to feed and water throughout the 42-day feeding period.

**Table 1:** Composition and nutrient levels of the basal diets (air-dry basis, %).

Item	Contents (%)
Ingredients	
Corn	73.00
Soybean meal	18.00
Wheat bran	5.00
Lysine	0.30
CaHPO_4_	0.50
Limestone	1.09
NaCl	0.40
Phytase 5,000 IU	0.04
Premix^a^	1.67
Total	100.00
Nutrient levels^b^	
DE, MJ/kg	14.20
Crude protein	16.12
Crude fiber	3.66
Ether extract	3.91
Total lysine	1.03
Total Ca	0.62
Total *P*	1.03

DE, digestible energy. ^a^Premix provided the following per kilogram of the diet: Vitamin A, 9,750.00 IU; Vitamin D3, 3,000.00 IU; Vitamin E, 24.00 mg; Vitamin K3, 3.00 mg; Vitamin B1, 3.00 mg; Vitamin B2, 7.50 mg; Vitamin B6, 4.50 mg; Vitamin B12, 30.00 μg; Nicotinamide, 36.00 mg; D-Calcium Pantothenate, 21.00 mg; Folic acid, 1.50 mg; Biotin, 0.15 mg; Fe (as ferrous sulfate), 180.00 mg; Cu (as copper sulfate), 108.75 mg; Mn (as manganese oxide), 59.63 mg; Zn (as zinc oxide), 50.00 mg; I (as potassium iodide), 1.05 mg; Se (as sodium selenite), 0.53 mg. ^b^DE was a calculated value, while the remaining values were determined via direct measurement.

### 2.3 Growth performance analysis

At the beginning and end of the trial, pigs in each replicate (pen) were weighed after overnight fasting (12 h), and the daily feed intake for each pen was recorded. These data were used to calculate the average daily gain (ADG), average daily feed intake (ADFI), and feed conversion ratio (F/G). Feed samples were collected and stored at −20 °C for chemical analysis.

### 2.4 Sample collection

Fresh fecal samples were collected from each pen on days 38–40 of the experiment. After collection, the samples were immediately placed into sample bags containing 10% dilute hydrochloric acid and then stored at −20 °C for subsequent analysis. After a 12-h overnight fast, six pigs per treatment were selected (one per replicate) for blood collection via jugular venipuncture. The collected blood was placed into anticoagulant-free tubes and centrifuged at 4,000 × *g* for 15 min at 4 °C. The serum (supernatant) was harvested and stored at −80 °C until subsequent analysis. Subsequently, the pigs were electrically stunned, exsanguinated, and slaughtered in an abattoir. The fresh *longissimus dorsi* (LD) muscle from the 6th to 7th ribs was harvested and rapidly preserved at 4 °C until subsequent analysis. Subcutaneous fat samples were isolated and rapidly fixed in 4% formalin for histopathological analysis of adipose tissue. The remaining muscle and adipose tissues were collected, packaged into cryovials, and snap-frozen in liquid nitrogen. These samples were later preserved at −80 °C until real-time PCR analysis. Jejunal, ileal, and colon chyme were obtained, then immediately banked at −80 °C until digestive enzyme concentration and 16S rRNA sequencing testing.

### 2.5 Serum biochemical index assays

Total protein (TP), glucose (GLU), triglycerides (TG), low-density lipoprotein cholesterol (LDL-C), high-density lipoprotein cholesterol (HDL-C), total cholesterol (TC), and blood urea nitrogen (BUN) concentrations were assessed using a Hitachi 7600 automatic biochemical analyzer (Hitachi, Tokyo, Japan).

### 2.6 Nutrient apparent digestibility measurements

Feed and fecal samples were ground into a powder and passed through a 40-mesh sieve before testing. The samples were analyzed for dry matter (DM; method 930.15), crude protein (CP; *N* × 6.25, method 984.13), ether extract (EE; method 920.39), and crude fiber (CF; method 978.10), according to AOAC (2007) (16). Gross energy (GE) was determined using an SDAC-6000 automatic calorimeter (Hunan Sundy Technology Co., Ltd., China). Acid-insoluble ash (AIA) was used as an endogenous indicator to determine apparent total tract digestibility (ATTD). The equation for ATTD calculation (%): 100—AIA content in diet/AIA content in feces × Nutrient content in feces/Nutrient content in diet × 100.

### 2.7 Enzyme concentration measurements

Trypsin and lipase concentrations in jejunal and ileal chyme were assayed using swine-specific enzyme-linked immunosorbent assay (ELISA) kits according to the manufacturer's protocol (Jiangsu Meimian Industrial Co., Ltd., Yancheng, China).

### 2.8 Meat quality analysis

The pH, meat color, drip loss, marbling score, and IMF concentration in the LD muscle were determined to evaluate meat quality. The pH values at 45 min and 24 h after slaughter were determined using a portable pH meter (pH-STAR; SFK Technology, Denmark). Meat color [redness (a^*^), yellowness (b^*^), and lightness (L^*^)] at 45 min and 24 h after slaughter was assayed using a Minolta CR-410 colorimeter (Osaka, Japan). The marbling score, meat color score, drip loss, and IMF content were analyzed as previously described (17).

### 2.9 Measurements of *longissimus dorsi* muscular fatty acid profiles

Total lipids were extracted from the LD samples (approximately 2.0 g) using a mixture of chloroform and methanol (2:1; v/v) according to the Folch method. The samples were extracted with petroleum ether for 4 h. The extracted fat was transferred into a hydrolysis tube, and 4 ml of isooctane was added to fully dissolve the sample. Subsequently, 0.7 ml (10 mol/L) potassium hydroxide and 5.3 ml methanol were supplemented, incubated at 55 °C for 1 h, and oscillated for 20 min. Then, 0.58 ml of 10 mol/L sulfuric acid was added and incubated again at 55 °C for 90 min with oscillation. After cooling, the mixture was mixed with n-hexane, allowed to stand for 5 min, and then centrifuged at 5 °C, 4,000 × g for 5 min. Subsequently, the supernatant was harvested, and the samples were analyzed using an Agilent 7890A GC-MS system (Agilent Technologies Inc., CA, USA).

### 2.10 Adipose tissue histology assays

Small segments of subcutaneous fat tissue were dehydrated in ascending grades of ethanol, cleared in xylene, embedded in paraffin, and sectioned into 5-μm-thick slices, which were then affixed and stained with hematoxylin and eosin (H&E) using a standard staining protocol. Images of the H&E-stained adipose tissue sections were captured using a Leica DM3000 microscope (Leica, Heidelberg, Germany). The total adipocyte area and the total number of adipocytes in each image were quantified using Image-Pro Plus 6.0 software (Media Cybernetics, MD, USA). The average adipocyte area was calculated by dividing the total adipocyte area by the total number of adipocytes.

### 2.11 mRNA expression of lipid metabolism-related genes

Total RNA from subcutaneous fat and muscle tissues was extracted using TRIzol reagent Invitrogen (Carlsbad, CA, USA), according to the manufacturer's instructions. RNA was quantified using a NanoDrop spectrophotometer (ND-2000; NanoDrop Technologies, Wilmington, DE, USA). The OD260/OD280 ratio ranging from 1.8 to 2.0 was considered acceptable. Reverse transcription was performed using an Evo M-MLV Reverse Transcription Kit (Accurate Biotechnology Co., Ltd., Changsha, China) following the manufacturer's instructions. RT-qPCR was performed using a Roche LightCycler^®^ 480 II system (SYBR^®^ Premix Ex Taq^®^ II, Takara, Kusatsu, Shiga, Japan), under the following cycling conditions: 30 s at 95 °C, followed by 40 cycles of 95 °C for 5 s and 60 °C for 30 s with primers for target genes. Primers were developed using Primer 5.0 software (Premier Biosoft International, Palo Alto, CA, USA) based on the gene sequence data from pigs (http://www.ncbi.nlm.nih.gov/genbank/) to yield the amplification products (Table 2). The 2^−Δ*ΔCt*^ method using β*-actin* as the housekeeping gene was used to calculate the relative gene expression.

**Table 2:** Primers used for quantitative real-time PCR.

Target genes	Primer sequences (5′-3′)	Gene bank
β-Actin	F: CTACGCCAACACGGTGCTGTC	XM_003357928.4
	R: CTCCTGCTTGCTGATCCACATCTG	
*FAS*	F: CTACCTTGTGGATCACTGCATAGA	NM_001099930.1
	R: GGCGTCTCCTCCAAGTTCTG	
*ACC*	F: TTCCAGGCACAGTCCTTAGG	XM_021066230.1
	R: TCATCCAACACGAGCTCAGT	
*CPT-1B*	F: ATGGTGGGCGACTAACT	XM_021091195.1
	R: TGCCTGCTGTCTGTGAG	
*PGC-1α*	F: GCCCTCATTTGATGCACTG	XM_021100441.1
	R: AGCTGAGTGTTGGCTGGTG	
*FATP1*	F: GGAGTAGAGGGCAAAGCAGG	XM_021076140.1
	R: AGGTCTGGCGTGGGTCAAAG	
*CD36*	F: CTGGTGCTGTCATTGGAGCAGT	XM_021102278.1
	R: CTGTCTGTAAACTTCCGTGCCTGTT	
*PPARγ*	F: AGGGCCAAGGATTCATGACA	XM_013981980.2
	R: GTGGTTCAACTTGAGCTGCA	
*LPL*	F: CACATTCACCAGAGGGTC	NM_214286.1
	R: TCATGGGAGCACTTCACG	
*SREBP1C*	F: GCGACGGTGCCTCTGGTAGT	NM_214157.2
	R: CGCAAGACGGCGGATTTA	

F, forward primer; R, reverse primer. FAS, fatty acid synthase; ACC, acetyl-CoA carboxylase; CPT-1B, carnitine palmitoyl transferase-1B; PGC-1α, peroxisome-proliferator-activated receptor γ coactivator-1α; FATP1, fatty acid binding protein 1; CD36, cluster of differentiation 36/fatty acid translocase; PPARγ, peroxisome proliferator-activated receptor γ; LPL, lipoprotein lipase; SREBP-1c, sterol regulatory element binding protein-1c.

### 2.12 16S rDNA sequencing of gut microbiota

Microbial genomic DNA was obtained from colonic content using the E.Z.N.A. Soil DNA Kit (Omega Biotek, Inc., Norcross, GA, USA) following the manufacturer's instructions. The purity and content of the extracted DNA were evaluated using 1% agarose gel electrophoresis. The V3-V4 hypervariable region of the bacterial 16S rRNA gene was amplified using primers 338F 5′-ACTCCTACGGGAGGCAGCAG3′) and 806R 5′-GGACTACHVGGGTWTCTAAT3′) with a 7300 Real-Time PCR system (ABI, CA, USA). The purified amplicons were paired-end sequenced (2 × 300 bp) on an Illumina MiSeq platform (Illumina, San Diego, CA, USA) by Novogene Co., Ltd. (Beijing, China). The NovaSeq 6000 software (Illumina, San Diego, CA, USA) was used to filter the obtained sequences from the samples. High-quality sequences were clustered into operational taxonomic units (OTUs) with a cutoff of 97% similarity using QIIME2 software (version QIIME2-202202, Northern Arizona University, Flagstaff, AZ, USA), and taxonomic annotation was performed against the Silva database (v138). Alpha diversity, beta diversity, and species abundance of the colonic microbiota were calculated at the OTU level. Alpha diversity indices (Shannon index, Ace index, Simpson index, Chao index, and Sobs index) were analyzed using QIIME2 software. Beta diversity was assessed using Bray–Curtis dissimilarity with principal coordinate analysis (PCoA) for visualization and ANOSIM for statistical validation. Statistical tests for differentially abundant taxa were performed using the linear discriminant analysis (LDA) effect size (LEfSe) method with default parameters (LDA score >2.5 and *P* < 0.05). Spearman correlation analysis was applied to evaluate the relationship between microbial species and lipid metabolism-related genes. The data were analyzed using the free online NovaSeq Cloud Platform (Novogene, Beijing, China) (https://magic.novogene.com).

### 2.13 Statistical analysis

A completely randomized design was used in this study. Before statistical analysis, all data were checked for normality using the Shapiro–Wilk test. Data (growth performance, serum biochemical parameters, meat quality, apparent nutrient digestibility, enzyme activity, and related molecular indicators) were analyzed by an independent-samples *t*-test using SPSS 27 statistical software (IBM Corporation, Armonk, NY, USA). Microbial differential analysis (based on relative abundance) was performed using the Kruskal–Wallis rank-sum test, and differential abundance of microbial taxa was further assessed using LEfSe analysis. Pearson's correlation analyses were conducted to explore the associations between gut microbiota and lipogenic gene expression. Results are presented as the mean ± standard error of the mean (SEM). Differences were considered significant at *P* < 0.05 and extremely significant at *P* < 0.01. Values with 0.05 ≤ *P* < 0.10 were regarded as a trend.

## 3 Results

### 3.1 Growth performance

As shown in Table 3, compared to the CON group, dietary PS supplementation significantly increased ADG (*P* < 0.05). However, there were no significant differences between the CON and PS groups in the final body weight, ADFI, or F/G (*P* > 0.05).

**Table 3:** Effect of dietary phytosterol supplementation on growth performance of the finishing pigs (*n* = 6)^a^.

Items	CON	PS	*P*-value
IBW, kg	73.98 ± 0.76	73.94 ± 1.51	0.98
FBW, kg	115.17 ± 1.78	119.71 ± 1.89	0.82
ADG, kg/d	0.98 ± 0.02	1.09 ± 0.02	0.02
ADFI, kg/d	3.48 ± 0.11	3.64 ± 0.06	0.27
F/G	3.56 ± 0.11	3.35 ± 0.09	0.18

IBW, initial body weight; FBW, final body weight; BW, body weight; ADG, average daily gain; ADFI, average daily feed intake; F/G, ratio of ADFI to ADG. CON, basal diet; PS, basal diet supplemented with 300 mg/kg phytosterols. ^a^Results in tables are presented as mean ± SEM; n, the number of replicate pens per treatment.

### 3.2 Serum biochemical parameters

PS supplementation increased serum HDL-C levels (*P* < 0.05) and tended to increase TG (*P* = 0.09) and GLU (*P* = 0.07) levels compared to those in the CON group, while significantly decreasing serum TP levels (*P* < 0.05). However, the diet supplemented with PS had no effect on the serum levels of BUN, TC, and LDL-C in finishing pigs (*P* > 0.05, Table 4).

**Table 4:** Effects of dietary phytosterol supplementation on serum biochemistry parameters of finishing pigs (*n* = 6)^a^.

Items	CON	PS	*P*-value
TP, g/L	74.95 ± 2.66	67.05 ± 1.25	0.03
BUN, mmol/L	7.38 ± 0.19	6.98 ± 0.02	0.59
TG, mmol/L	0.64 ± 0.06	0.92 ± 0.13	0.09
TC, mmol/L	2.42 ± 0.06	2.38 ± 0.08	0.71
LDL-C, mmol/L	0.97 ± 0.03	0.93 ± 0.01	0.34
HDL-C, mmol/L	0.84 ± 0.03	0.96 ± 0.03	0.04
GLU, mmol/L	4.83 ± 0.52	6.20 ± 0.44	0.07

TP, total protein; BUN, serum urea nitrogen; TG, triglycerides; TC, total cholesterol; GLU, glucose; LDL-C, low-density lipoprotein cholesterol; HDL-C, high-density lipoprotein cholesterol. CON, basal diet; PS, basal diet supplemented with 300 mg/kg phytosterols. ^a^Results in tables are presented as mean ± SEM; n, number of pigs per treatment.

### 3.3 Nutrient apparent digestibility

The ATTD of nutrients is shown in Table 5. Compared to the CON group, supplementation with PS significantly increased the apparent digestibility of EE and CF (*P* < 0.01). However, PS had no effect on DM, CP, or GE digestibility (*P* > 0.05).

**Table 5:** Effect of dietary phytosterol supplementation on nutrient apparent digestibility in finishing pigs (%, as-fed basis, *n* = 6)^a^.

Items	CON	PS	*P*-value
DM, %	95.69 ± 0.09	95.77 ± 0.16	0.72
CP, %	85.33 ± 0.78	86.08 ± 0.88	0.55
EE, %	76.11 ± 2.04	82.49 ± 0.67	0.04
CF, %	38.58 ± 0.18	49.59 ± 1.76	< 0.01
GE, %	87.55 ± 0.28	88.05 ± 0.38	0.35

DM, dry matter; CP, crude protein; CF, crude fiber; EE, ether extract; GE, total energy. CON, basal diet; PS, basal diet supplemented with 300 mg/kg phytosterols. ^a^Results in tables are presented as mean ± SEM; n, the number of replicate pens per treatment.

### 3.4 Digestive enzyme concentration

As shown in Table 6, PS supplementation increased lipase concentration in the jejunum (*P* < 0.01) and decreased jejunal trypsin concentration compared to the CON group (*P* < 0.01). No significant differences were detected in ileal lipase and trypsin concentrations between the CON and PS groups (*P* > 0.05).

**Table 6:** Effects of dietary phytosterol supplementation on digestive enzyme concentrations in finishing pigs (*n* = 6)^a^.

Items, ng/ml	CON	PS	*P*-value
Jejunum			
Trypsin	1,811.52 ± 122.91	1,284.50 ± 75.60	< 0.01
Lipase	189.77 ± 9.41	215.75 ± 3.13	< 0.01
Ileum			
Trypsin	1,621.85 ± 114.85	1,483.39 ± 152.40	0.49
Lipase	192.09 ± 14.10	208.44 ± 16.68	0.47

CON, basal diet; PS, basal diet supplemented with 300 mg/kg phytosterols. ^a^Results in tables are presented as mean ± SEM; n, number of pigs per treatment.

### 3.5 Meat quality

As shown in Table 7, dietary PS supplementation significantly improved the IMF content and pH_45min_ value in the LD muscle compared with the CON group (*P* < 0.05). In contrast, no differences were observed in the other meat quality parameters between the CON and PS groups (*P* > 0.05).

**Table 7:** Effects of dietary phytosterol supplementation on meat quality of finishing pigs (*n* = 6)^a^.

Items	CON	PS	*P*-value
pH_45min_	6.43 ± 0.07	6.69 ± 0.05	0.02
pH_24h_	5.69 ± 0.02	5.74 ± 0.06	0.49
L^*^ 45 min	43.34 ± 0.69	44.29 ± 0.18	0.31
a^*^ 45 min	6.49 ± 0.52	5.59 ± 0.98	0.63
b^*^ 45 min	2.86 ± 0.18	3.03 ± 0.47	0.74
L^*^ 24 h	46.61 ± 1.60	51.02 ± 1.90	0.10
a^*^ 24 h	8.82 ± 0.59	7.39 ± 0.81	0.18
b^*^ 24 h	5.32 ± 0.57	5.83 ± 0.65	0.57
Meat color scores	3.50 ± 0.01	3.30 ± 0.14	0.23
Drip loss, %	2.05 ± 0.32	2.15 ± 0.15	0.78
Marbling scores	3.25 ± 0.11	3.33 ± 0.15	0.67
IMF, %	1.97 ± 0.02	2.65 ± 0.17	0.01

L^*^, lightness; a^*^, redness; b^*^, yellowness; IMF, intramuscular fat. CON, basal diet; PS, basal diet supplemented with 300 mg/kg phytosterols. ^a^Results in tables are presented as mean ± SEM; n, number of pigs per treatment.

### 3.6 Muscular fatty acid composition

As shown in Table 8, compared to the CON group, PS supplementation increased total fatty acid concentrations in LD muscle, such as C14:0, C17:1, C18:2n6c, C18:3n3, and n-3 PUFA (*P* < 0.05). However, PS had no effects on other fatty acid concentrations in the LD muscle.

**Table 8:** Effects of dietary phytosterol supplementation on fatty acid profile in *longissimus dorsi* muscle of finishing pigs (*n* = 6)^a^.

Fatty acids, μg/g	CON	PS	*P-*value
C14:0	136.54 ± 22.64	225.37 ± 27.24	0.04
C14:1	3.08 ± 0.44	2.42 ± 0.12	0.39
C15:0	6.41 ± 0.11	7.08 ± 1.00	0.25
C15:1	8.68 ± 1.32	7.01 ± 0.25	0.23
C16:0	3,784.93 ± 433.02	4,739.48 ± 483.86	0.20
C16:1	597.99 ± 19.01	644.46 ± 17.39	0.15
C17:0	33.48 ± 2.76	38.73 ± 3.23	0.28
C17:1	24.52 ± 2.33	33.85 ± 2.69	0.04
C18:0	1,798.99 ± 211.85	2,213.90 ± 278.08	0.27
C18:1n9c	6,351.11 ± 374.85	6,904.06 ± 355.01	0.34
C18:1n9t	28.82 ± 1.49	26.72 ± 1.59	0.38
C18:2n6c	1,307.66 ± 42.43	1,554.78 ± 48.14	0.01
C18:3n6	37.86 ± 0.89	39.28 ± 1.32	0.39
C18:3n3	26.78 ± 0.92	36.96 ± 1.13	< 0.01
C20:0	30.25 ± 1.92	33.75 ± 1.53	0.24
C20:1n9	105.72 ± 18.65	127.65 ± 17.16	0.44
C20:2	36.35 ± 1.53	40.38 ± 1.33	0.18
C21:0	17.33 ± 1.08	16.91 ± 0.29	0.48
C20:3n6	37.31 ± 1.08	38.07 ± 1.94	0.72
C20:4n6	226.83 ± 5.27	231.11 ± 4.25	0.57
C20:3n3	21.07 ± 0.62	19.76 ± 0.54	0.22
C22:0	16.87 ± 0.36	16.59 ± 0.37	0.65
C20:5n3	17.89 ± 0.13	17.90 ± 0.35	0.98
C22:1n9	18.55 ± 0.46	18.07 ± 0.46	0.54
C24:0	19.81 ± 0.37	19.36 ± 0.34	0.46
C24:1n9	18.01 ± 0.21	18.15 ± 0.35	0.73
SFA^b^	6,957.56 ± 69.30	6,856.73 ± 82.23	0.40
MUFA^c^	7,124.35 ± 398.85	7,777.72 ± 394.54	0.30
PUFA^d^	1,729.69 ± 20.52	1,700.19 ± 39.93	0.50
PUFA/SFA	0.26 ± 0.02	0.26 ± 0.03	0.98
n-3 PUFA^e^	65.61 ± 1.17	74.63± 1.96	< 0.01
n-6 PUFA^f^	1,766.42 ± 75.00	1,825.18 ± 51.25	0.63
n-6/n-3 PUFA	35.22 ± 0.85	32.25 ± 1.59	0.18

SFA, saturated fatty acid; MUFA, monounsaturated fatty acid; PUFA, polyunsaturated fatty acid. CON, basal diet; PS, basal diet supplemented with 300 mg/kg phytosterols. ^a^Results in tables are presented as mean ± SEM; n, number of pigs per treatment. ^b^SFA = C14:0 + C15:0 + C16:0 + C17:0 + C18:0 + C20:0 + C22:0 + C23:0 + C24:0. ^c^MUFA = C14:1 + C16:1 + C18:1n9c + C18:1n9t + C20:1 + C24:1. ^d^PUFA = C18:2n6c + C18:3n6 + C18:3n3 + C20:2 + C20:3n6 + C20:4n6 + C20:3n3 + C22:6n3. ^e^n-3 PUFA = C18:3n3 + C20:3n3 + C20:5n3. ^f^n-6 PUFA = C18:2n6c + C20:3n6 + C20:4n6.

### 3.7 H&E staining of adipose tissue

**Figure 1:**
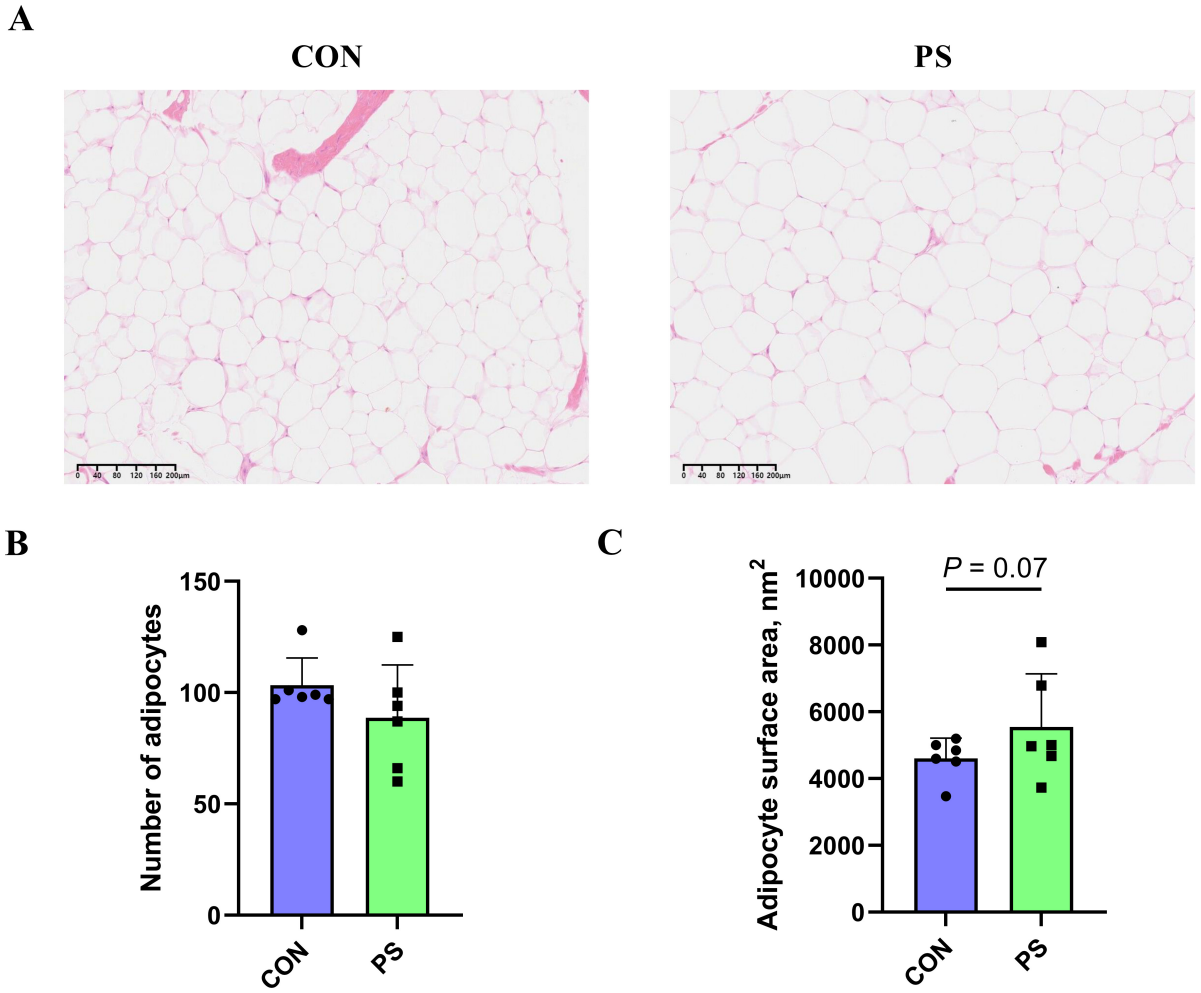
Effect of dietary phytosterol supplementation on subcutaneous adipose tissue morphology of finishing pigs (*n* = 6). **(A)** Adipocyte morphology. **(B)** Number of adipocytes. **(C)** Adipocyte surface area. CON, basal diet; PS, basal diet supplemented with 300 mg/kg phytosterols. Values with 0.05 ≤ *P* < 0.10 were regarded as a trend. Results on the column chart are expressed as the mean ± SEM; *n* = 6, number of pigs per treatment.

To explore how PS is involved in lipid deposition, histological analysis of subcutaneous fat was performed. As shown in Figure 1, the adipocytes in the PS group had a uniform size, clear borders, and tight arrangement. Within the same visual field, the number of adipocytes in the PS group was not significantly different (*P* > 0.05) from that in the CON group. However, the adipocyte surface area tended to increase in the PS group (*P* = 0.07).

### 3.8 mRNA expression of lipid metabolism-related genes

**Figure 2:**
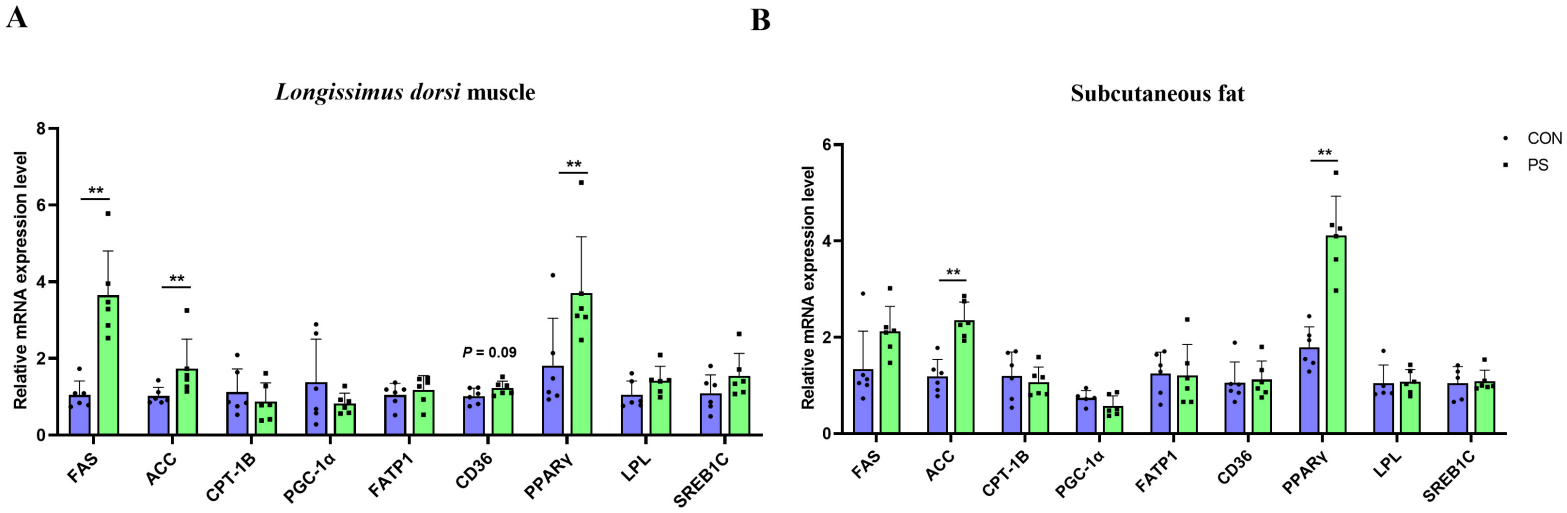
Effects of dietary phytosterol supplementation on the mRNA expression of lipid metabolism-related genes in finishing pigs (*n* = 6). **(A)** mRNA expression of lipid metabolism-related genes in *the longissimus dorsi* muscle. **(B)** mRNA expression of lipid metabolism-related genes in subcutaneous fat. *FAS*, fatty acid synthase; *ACC*, acetyl-CoA carboxylase; *PPAR*γ, peroxisome proliferator-activated receptor γ; *SREBP-1c*, sterol regulatory element binding protein-1c; *LPL*, lipoprotein lipase; *CPT-1B*, carnitine palmitoyl transferase-1B; *PGC-1*α, peroxisome proliferator-activated receptor γ coactivator-1α; *CD36*, cluster of differentiation 36/fatty acid translocase; *FATP1*, fatty acid-binding protein 1. CON, basal diet; PS, basal diet supplemented with 300 mg/kg phytosterols. **Values with *P* < 0.01, 0.05 ≤ *P* < 0.10 were regarded as trends. Results in the column chart are expressed as the mean ± SEM; *n* = 6, number of pigs per treatment.

To further investigate the association between PS and fat deposition in the muscle and subcutaneous fat, the expression of lipid metabolism-related genes was detected. As shown in Figure 2A, PS upregulated the mRNA expression of *FAS, ACC*, and *PPAR*γ in the LD muscle (*P* < 0.05) and tended to upregulate the expression of CD36 (*P* = 0.09) compared with the CON group. Additionally, PS had no significant effect on the mRNA expression of *CPT-1B, PGC-1*α*, FATP1, LPL*, or *SREBP1c (P* > 0.05).

In subcutaneous fat (Figure 2B), pigs fed the PS-supplemented diet had higher *ACC* and *PPAR*γ mRNA levels than those fed the CON diet (*P* < 0.01). Likewise, PS did not modulate the mRNA expression of other lipid metabolism-related genes in subcutaneous fat.

### 3.9 Gut microbial diversity and community

**Figure 3:**
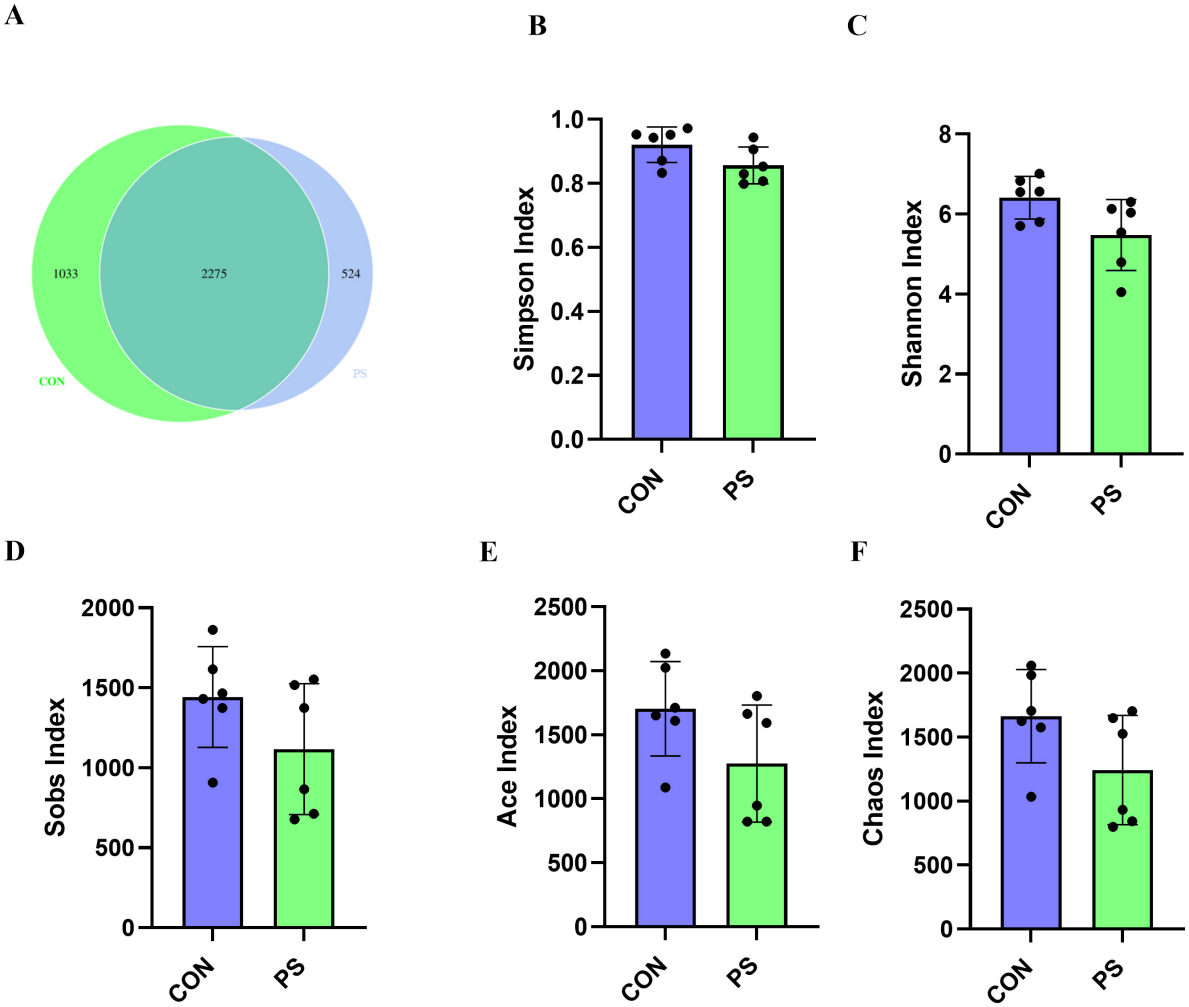
Effects of dietary phytosterol supplementation on colonic microbial OTUs and alpha diversity (*n* = 6). **(A)** Common and unique OTUs between the CON and PS groups. **(B)** Simpson index. **(C)** Shannon index. **(D)** Sobs index. **(E)** Ace index. **(F)** Chao index. CON, basal diet; PS, basal diet supplemented with 300 mg/kg phytosterols. Results on the column chart are expressed as the mean ± SEM; *n* = 6, number of pigs per group.

**Figure 4:**
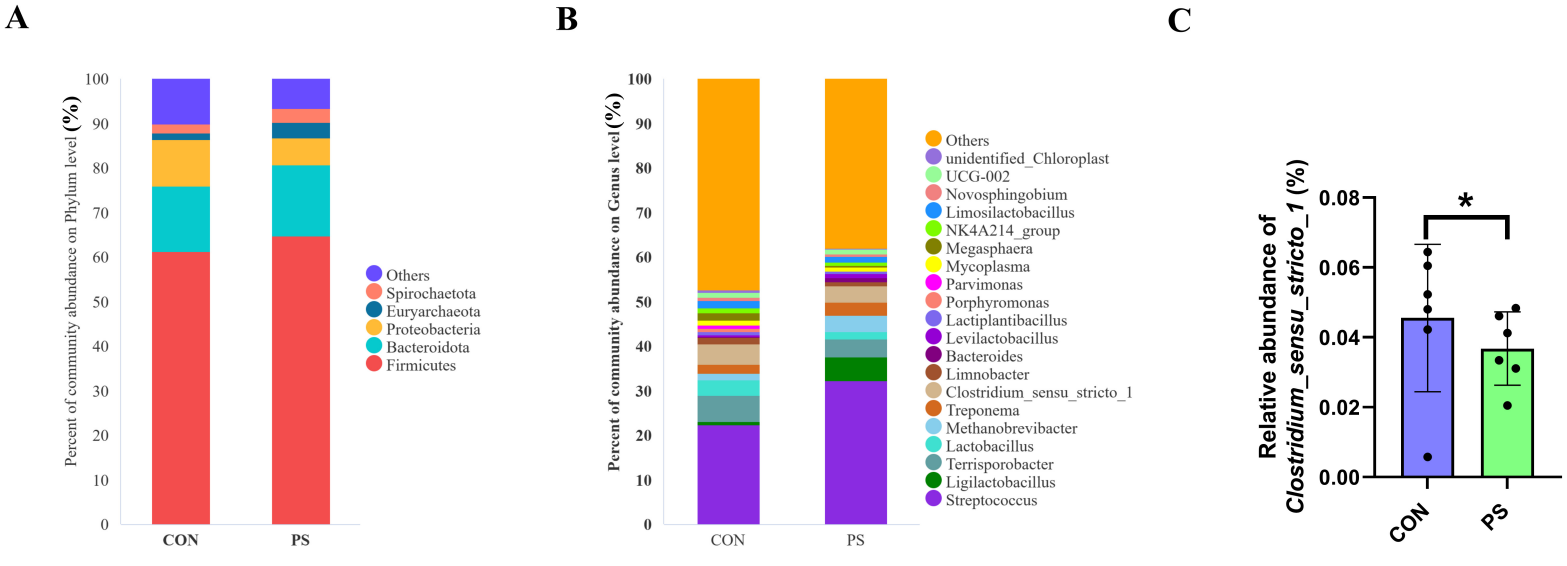
Effects of dietary phytosterol supplementation on colonic microbial phyla and genera (*n* = 6). **(A)** Microbial phylum composition. **(B)** Microbial genus composition. **(C)** Relative abundance of *Clostridium_sensu_stricto_1*. CON, basal diet; PS, basal diet supplemented with 300 mg/kg phytosterols. *P < 0.05. Results on the column chart are expressed as the mean ± SEM; *n* = 6, number of pigs per treatment.

A total of 3,832 OTUs were identified in the two groups. The number of common OTUs was 2,275, and the number of unique OTUs in the CON and PS groups were 1,033 and 524, respectively (Figure 3A). The alpha-diversity indices covering Simpson, Shannon, Sobs, Ace, and Chao are summarized in Figures 3B–F; no significant differences in the abundances and diversities of the colonic microbial community were observed between the CON and PS groups. Bar plots of microbiota phyla showed that Bacteroidetes and Firmicutes were the dominant phyla in finishing pigs from both the CON and PS groups, followed by Proteobacteria (Figure 4A), and the relative abundance of Firmicutes and Bacteroidetes increased by 3.37 and 1.35% after PS treatment, respectively. At the genus level, the top five predominant genera were *Streptococcus* (32.36%), *Ligilactobacillus* (5.31%), *Terrisporobacter* (3.99%), *Clostridium_sensu_stricto_1* (3.67%), and *Methanobrevibacter* (3.52%; Figure 4B). In addition, dietary PS supplementation significantly decreased the relative abundance of *Clostridium_sensu_stricto_1* (*P* < 0.05, Figure 4C).

**Figure 5:**
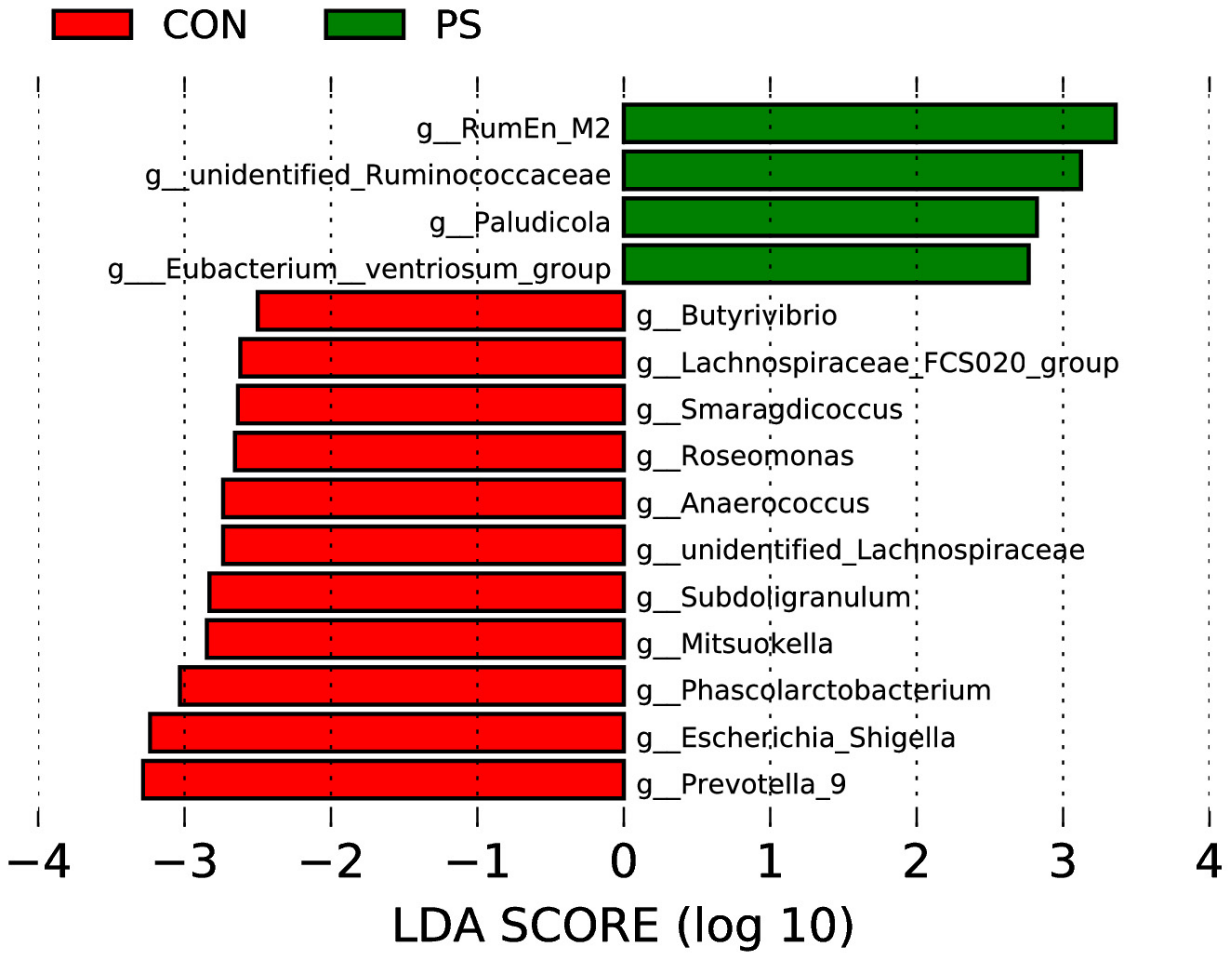
Histogram of linear discriminant analysis (LDA) scores computed for features differentially abundant in the PS-supplemented and CON groups (*n* = 6). CON, basal diet; PS, basal diet supplemented with 300 mg/kg phytosterols. Species with significant differences that have an LDA score greater than 2.5 are presented. *n* = 6, number of pigs per treatment.

The different abundances of overall microbes at the genus level were analyzed using LEfSe (LDA > 2.5, Figure 5). Compared to the CON group, supplementation with PS decreased the relative abundance of *Phascolarctobacterium, Escherichia-Shigella, Mitsuokella, Butyrivibrio, Lachnospiraceae FCS020, Roseomonas, Anaerococcus*, Smaragdicoccus, *unidentified Lachnospiraceae, Subdoligranulum*, and *Prevotella_9*, while enhancing the relative abundance of *Rum En_M2, unidentified Ruminococcaceae, Paludicola*, and *Eubacterium ventriosum*.

### 3.10 Correlation analysis of microbiota and lipid metabolism-related genes

**Figure 6:**
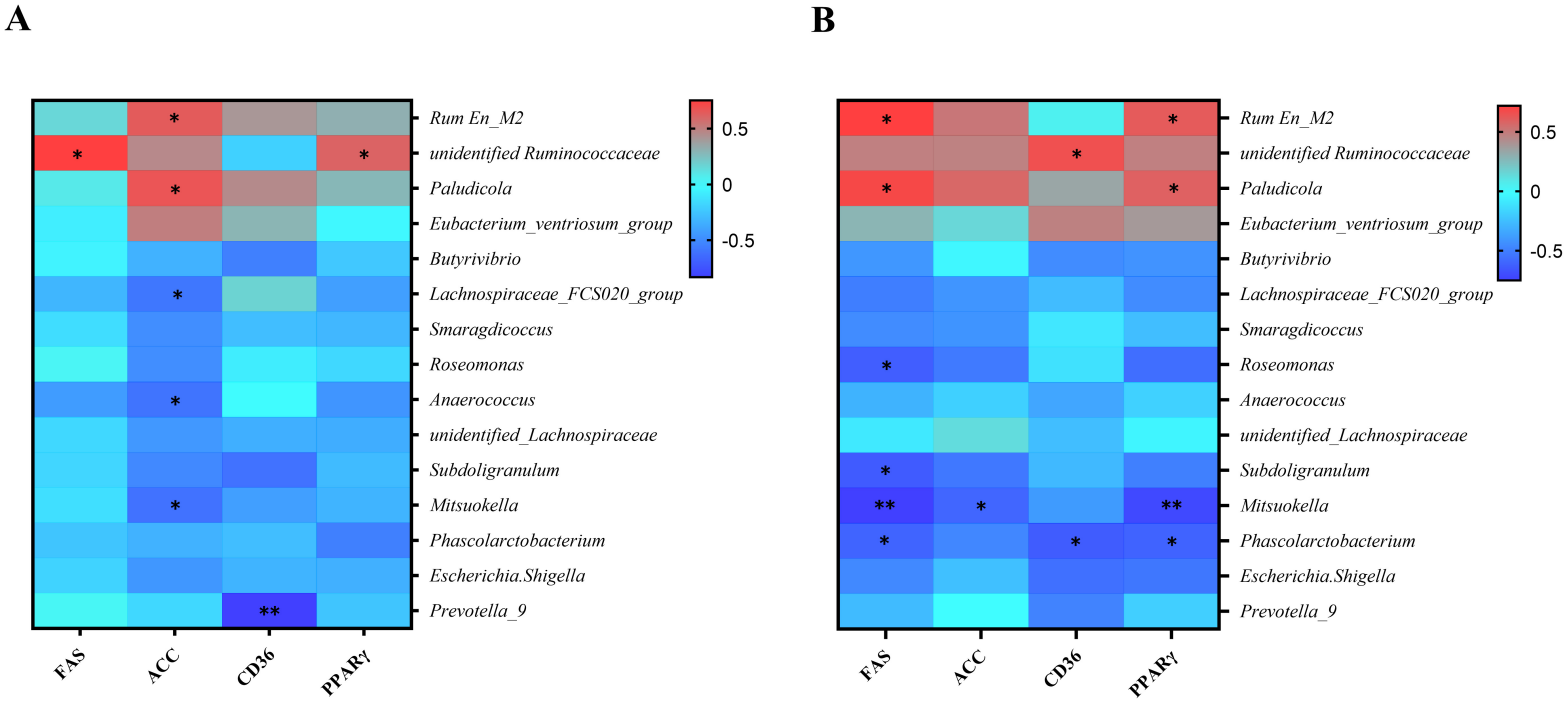
Correlation analysis between colonic microbiota and lipid metabolism-related gene expression (*n* = 6). **(A)** Heatmap of Spearman's correlation between colonic microbiota and lipid metabolism gene expression in the longissimus dorsi muscle. **(B)** Heatmap of Spearman's correlation between colonic microbiota and lipid metabolism gene expression in subcutaneous fat. *FAS*, fatty acid synthase; *ACC*, acetyl-CoA carboxylase; *CD36*, cluster of differentiation 36/fatty acid translocase; *PPAR*γ, peroxisome proliferator-activated receptor-γ. Colors ranging from blue to red represent the magnitude of the correlation. Significant correlations are noted as *P < 0.05; **P < 0.01. *n* = 6, number of pigs per treatment.

To further explore the correlations between gut microbiota and lipid metabolism in the *longissimus dorsi* muscle and subcutaneous fat, Spearman's correlation analysis was performed. As shown in Figure 6A, the abundances of *Rum En_M2, Paludicola*, and *unidentified Ruminococcaceae* were positively correlated with the lipogenesis-related genes in LD muscle such as *FAS, ACC, CD36*, and *PPAR*γ (*P* < 0.05). In contrast, the abundances of the *Lachnospiraceae FCS020 group, Anaerococcus, Mitsuokella*, and *Prevotella_9* were negatively correlated with lipogenic genes (*P* < 0.05, Figure 6A). The abundance of *Rum En_M2, Paludicola*, and *unidentified Ruminococcaceae* was positively correlated with lipogenic genes in subcutaneous fat (*P* < 0.05, Figure 6B). Moreover, significant negative relationships were observed between the abundance of *Mitsuokella, Roseomonas, Subdoligranulum*, and *Phascolarctobacterium* and lipogenic genes (*P* < 0.05, Figure 6B).

## 4 Discussion

Recently, plant extracts have attracted increasing attention as feed additives owing to their beneficial effects on growth performance and meat quality. PS, mainly derived from vegetable oils, legume products, fruit, and plant seeds, have been reported to exert a positive role in growth promotion, antioxidation, immune regulation, and lowering cholesterol (18). Accumulating data have demonstrated that PS can improve the growth performance of livestock and poultry by enhancing immune function, increasing antioxidant capacity, and improving intestinal morphology to reduce diarrhea rates (19, 20). Previous studies have reported that dietary PS supplementation increases FBW, ADG, and ADFI in finishing pigs (21). Similar positive effects of PS on ADG and feed conversion ratio have also been observed in broilers (22). Similarly, in this study, finishing pigs in the PS treatment group grew faster than those in the CON treatment group, indicating that dietary PS supplementation could improve growth performance. Notably, PS supplementation significantly increased the ADG of finishing pigs but did not significantly increase the FBW. This phenomenon may result from individual biological variation, limited trial duration, and altered *in vivo* energy partitioning, which limits the accumulation of daily growth advantages, resulting in no significant difference in FBW. Nevertheless, a notable limitation of this study is the evaluation of a single PS supplementation dose, which was selected based on the effective concentrations reported in previous studies (15). Due to the lack of multiple dietary gradients, a dose–response relationship could not be established, and the optimal dose of PS remains to be verified. This information is critical for practical feeding recommendations as it helps balance the efficacy and cost-effectiveness of swine production. Future studies should include multiple PS dose levels to determine the optimal inclusion concentration and clarify its safety range for finishing pigs.

The apparent digestibility of nutrients is closely associated with the degree of nutrient utilization by animals. Numerous studies have reported that β-sitosterol can improve the small intestinal mucosal surface area, thereby promoting the digestion and absorption of nutrients (23). In the current study, dietary addition of PS increased CF and EE digestibility. Similarly, research has reported that PS supplementation increases the apparent digestibility of CP and EE (24). Digestive enzyme activities in the intestine reflect the digestive metabolism level and nutrient utilization ability of the animal (25). Plant extracts have been demonstrated to stimulate endogenous digestive enzyme secretion, such as lipase (26). In this study, the addition of PS improved jejunal lipase concentration in finishing pigs. Similarly, supplementation with PS increased lipase and amylase activities in broilers (27). These results indicate that the improvement in growth performance after PS supplementation may be closely associated with increased digestive enzyme concentrations and enhanced nutrient digestibility.

Blood biochemical parameters reflect the health status and nutrient metabolism of animals (28). This study showed that PS supplementation increased serum GLU levels in finishing pigs. Serum TG concentration indicates lipid metabolism in the body (29). LDL transports cholesterol (CHOL) from the liver to peripheral tissues, whereas HDL promotes the transfer of CHOL from peripheral tissues to the liver for metabolism or bile excretion. Additionally, HDL can enhance the serum decomposition of very low-density lipoprotein (VLDL) and chylomicrons and inhibit the binding of LDL to cell receptors, thereby reducing CHOL absorption by peripheral tissues (30). PS are structurally similar to CHOL, thus affecting cholesterol absorption by competing with CHOL for micelles in the intestine (31). Previous studies in mice have shown that PS supplementation improves serum HDL levels (32). Consistent with these findings, we observed an increase in HDL-C and TG levels in the serum of finishing pigs after PS treatment, which may modulate lipid metabolism. Consistently, in adipose tissue, we found that the adipocyte surface area in the PS group tended to increase, although the adipocyte number in the PS group was not significantly different from that in the CON group. These results demonstrate that PS supplementation regulates lipid metabolism in finishing pigs.

pH is a key parameter for evaluating meat quality and is associated with color, tenderness, and water-holding capacity (33). Low pH values are associated with lactic acid accumulation from muscle glycolysis, which affects meat color and water-holding capacity (34). Conversely, an increase in the ultimate pH value exerts a beneficial effect on meat tenderness (35). It has been shown that PS can improve meat quality and flavor in finishing pigs. In this study, dietary supplementation with PS elevated the pH_45min_ value in the LD muscle. Our observation is consistent with previous studies where supplementation of PS elevated the pH value in the breast muscle of broilers (36). Additionally, we discovered that PS supplementation improved IMF content in the LD muscle, which is in line with previous studies (37). As is well-established, IMF content is a critical index of meat quality, which directly affects the flavor, juiciness, and tenderness of pork (38). Numerous studies have suggested that IMF content below 2.0%−2.5% may negatively affect the flavor and juiciness of pork (39). Consequently, the higher pH and IMF content in the LD muscle revealed that PS supplementation improved tenderness, flavor, and juiciness of the meat.

Furthermore, the fatty acid profile of muscle is crucial for flavor generation and nutritional value of pork (40). Saturation of fatty acids influences fat hardness and thereby impacts pork quality, with flavor normally being positively correlated with the MUFA ratio (41). However, excessive ingestion of saturated fatty acids (SFA) is thought to enhance the likelihood of obesity and cardiovascular disease (42). However, moderate intake of stearic acid has been shown to reduce serum CHOL levels (43). Notably, essential fatty acids (EFAs), particularly n-3 PUFAs, offer benefits to human and animal health by regulating metabolic syndrome and preventing cardiovascular diseases (44). Indeed, it is well-documented that a higher intake of n-3 PUFA or a suitable ratio of n-6 to n-3 PUFA can decrease the likelihood of inflammation and obesity (45). α-linolenic acid (C18:3n3), a typical C-18 n-3 PUFA, is vital for synthesis, metabolism, and conversion into essential DHA and EPA in the body (46). Increasing evidence suggests that α-linolenic acid protects against cardiovascular diseases and regulates systemic lipid metabolism (47). In this study, supplementation of PS improved the fatty acid composition in the LD muscle, importantly increasing the total fatty acid concentration, including C14:0, C17:1, C18:2n6c, C18:3n3, and n-3 PUFA, indicating that PS could improve the nutritional quality of pork from the perspective of fatty acid nutrition.

To investigate the mechanism by which PS promotes dietary lipid deposition, the mRNA expression of genes involved in lipid metabolism was analyzed. Lipid uptake is primarily mediated by fatty acid transporters, specifically *FATP1* and *FAT/CD36*. When overexpressed, the transport of fatty acids across cell membranes is normally increased (48). Previous studies have demonstrated that targeted upregulation of *CD36* expression could increase intestinal fatty acid absorption and lipid deposition (49). In this study, supplementation with PS upregulated *CD36* mRNA expression levels in the muscle to some extent, indicating that PS may be involved in the regulation of fatty acid transport in muscle tissue. *ACC* and *FAS*, as two key rate-limiting enzymes, are classic markers for *de novo* fatty acid synthesis (50). *PPAR*γ, as a critical transcription factor, participates in lipid synthesis and metabolism and regulates the expression of the downstream target genes *ACC* and *LPL* (51). Research has shown that PS can alter the expression of host lipogenic genes (52). Consistent with the above findings, our data demonstrated that supplementation with PS improved the mRNA expression levels of *FAS, ACC*, and *PPAR*γ in subcutaneous fat and LD muscle. As expected, the higher contents of IMF and some fatty acids in the muscle of the PS group are most likely attributed to heightened fatty acid uptake and synthesis driven by these upregulated genes.

Recent studies have indicated that the gut microbiota exerts a key effect on regulating host lipid metabolism (53). Bacteroidetes and Firmicutes are the most abundant phyla in the gut microbiota of humans and pigs. Concerning lipid metabolism, an increased abundance of Firmicutes has been reported to increase lipid synthesis and promote lipid deposition (54). Research by Dai et al. (22) on dietary PS-supplemented broilers demonstrated the upregulated abundance of Firmicutes and concomitantly caused a rise in abdominal fat deposition. In the current study, supplementation with PS elevated the colonic relative abundance of Firmicutes, which supports the results of previous investigations (55). These outcomes suggest that regulation of gut microbiota by PS is closely associated with the increased lipid deposition observed in finishing pigs. Subsequent analysis at the genus level demonstrated that PS reduced the abundance of *Clostridium_sensu_stricto_1*, a member of the genus Clostridium implicated in the development of intestinal mucosal inflammation (56). Furthermore, our LEfSe analysis revealed that supplementation with PS dramatically lowered the abundances of *Escherichia-Shigella*. *Escherichia-Shigella*, a common causative agent of bacterial diarrhea, damages tight junctions through interplays between secreted bacterial virulence factors and the intestinal epithelium (57). It has also been reported that *Escherichia-Shigella* can disrupt host hepatic lipid metabolism by upregulating lipid synthesis and suppressing hepatic lipid β-oxidation (58). These results suggest that PS may help protect the host from harmful intestinal pathogens by inhibiting their colonization.

Accumulating evidence indicates that *Prevotella* spp. can affect lipid metabolism and fat deposition in animals. For example, previous research has demonstrated that the relative abundance of *Prevotella* in the gut microbiota is strongly correlated with subcutaneous and intramuscular fat content (59). *Paludicola* is a new genus within the family *Ruminococcaceae*, whose function remains unknown (60). However, *Ruminococcaceae* is known to participate in regulating fatty acid and bile acid metabolism, processes that are highly involved in host obesity (50). Our results revealed that PS supplementation decreased the relative abundance of *Prevotella* and increased the abundance of *unidentified Ruminococcaceae* and *Paludicola species*. Notably, these bacteria were significantly correlated with the expression of lipogenic genes in the LD muscle and subcutaneous fat. Recent studies have revealed that gut microbiota metabolites, particularly short-chain fatty acids, serve as crucial signaling molecules that can regulate host lipogenic genes, such as *PPAR*γ and *CD36* (61). Therefore, we infer that PS-induced reshaping of the gut microbiota may alter the production of specific microbial metabolites, which subsequently upregulate key lipid-metabolism genes, enhancing IMF synthesis and improving meat quality.

## 5 Conclusions

In conclusion, dietary PS supplementation is effective in improving the growth performance and meat quality of finishing pigs. The beneficial effects are accompanied by increased apparent digestibility of nutrients and IMF deposition, which are associated with the regulation of gut microbiota and lipid metabolism. Furthermore, PS administration upregulates the expression of lipogenesis-related genes, which could improve the meat fatty acid profile, markedly increasing the EFA content and providing health benefits to consumers.

## Data Availability

The original contributions presented in the study are publicly available.The 16S rRNA sequencing data have been deposited in the NCBI BioProject database under the accession number PRJNA1457271.
